# IgE And IgG Antibody Responses to α‐Gal and Association With Carotid Stenosis in Swedish Patients

**DOI:** 10.1111/all.70329

**Published:** 2026-04-12

**Authors:** Marija Perusko, Danijela Apostolovic, Jeanette Grundström, Malin Kronqvist, Mirjana Radomirovic, Anna Smed‐Sörensen, Ulf Hedin, Marianne van Hage

**Affiliations:** ^1^ Division of Immunology and Respiratory Medicine, Department of Medicine Solna Karolinska Institutet and Karolinska University Hospital Solna Sweden; ^2^ Center for Molecular Medicine Karolinska University Hospital Solna Sweden; ^3^ Department of Molecular Medicine and Surgery Karolinska Institutet and Karolinska University Hospital Sweden; ^4^ University of Belgrade – Faculty of Chemistry Belgrade Serbia

To the Editor,

IgE sensitization to the oligosaccharide galactose‐α‐1,3‐galactose (α‐Gal), acquired through tick bites, is well established as cause of delayed allergic reactions to mammalian meat [1]. Emerging evidence suggests that α‐Gal sensitization may also be implicated in coronary artery disease. Increased atheroma burden and plaques with more unstable features were associated with IgE to α‐Gal in patients undergoing intravascular ultrasound or CT coronary angiography in tick‐endemic areas in the United States and Australia [2, 3]. In addition, high levels of IgG3 to α‐Gal have also been related to increased atheroma burden [4]. The hypothesis is that, in individuals with elevated IgE and/or IgG against α‐Gal, dietary α‐Gal‐containing glycolipids carried by low‐density lipoproteins may trigger subclinical activation of mast cells and/or promote foam cell formation within atherosclerotic plaques, thereby contributing to the development of atherosclerosis [2, 5].

This study constitutes the first European investigation of the clinical relevance of IgE and IgG antibodies to α‐Gal in a cohort of patients with carotid atherosclerosis. In 100 patients from the Biobank of Karolinska Endarterectomies (BiKE), undergoing stroke‐preventive surgery (carotid endarterectomy; CEA) for carotid stenosis, we measured total IgE as well as IgE and IgG to α‐Gal (ImmunoCAP o215; Thermo Fisher Scientific; cutoff ≥ 0.1 kU_A_/L and 2 mg/L, respectively). A control cohort of 27 subjects without cardiovascular disease was also included in the analyses (for details, see Table S1). Written informed consent was obtained from all participants. Statistical analysis is described in the Supporting Information.

The prevalence of IgE sensitization to α‐Gal was 18% in BiKE and 15% in the control cohort (*p* > 0.999, Figure 1A). The sensitization rate to α‐Gal substantially exceeded that reported for other common food allergens [6]. There was no significant difference in α‐Gal‐specific IgE levels between the two cohorts (*p* = 0.593, Figure 1B). However, α‐Gal‐specific IgG concentrations were higher in BiKE than in the control cohort (*p* = 0.024, Figure 1C). The 95% confidence interval for IgG concentrations was 2.74–3.94 in BiKE and 2.00–3.46 in the control cohort, indicating higher overall levels in patients and a possible association with the disease.

**FIGURE 1:**
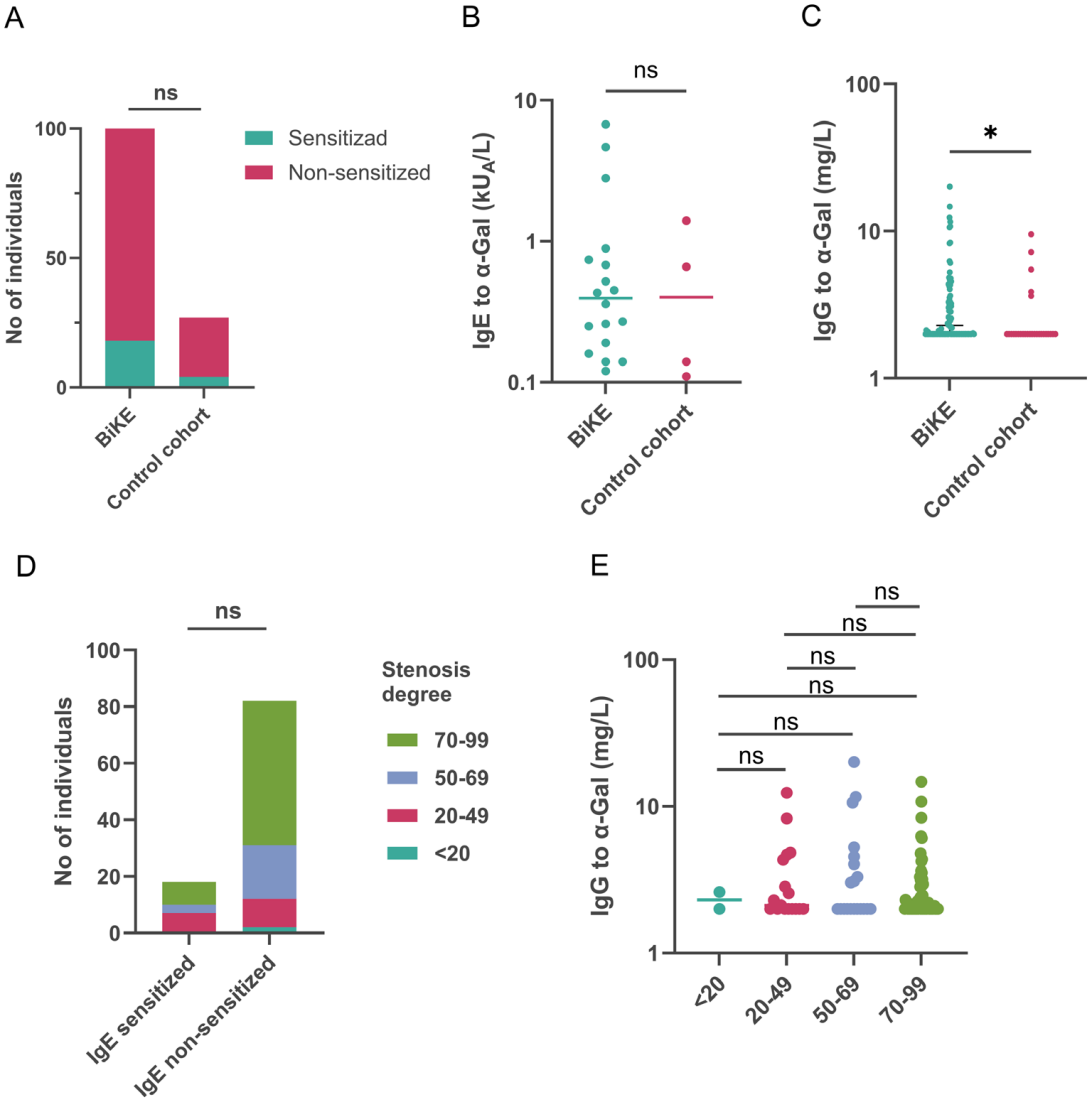
Comparison between cohort of patients with carotid stenosis (BiKE) and control cohort for (A) prevalence of IgE sensitization to α‐Gal (Fisher's exact test), (B) IgE levels to α‐Gal (Mann–Whitney test), and (C) IgG levels to α‐Gal (Mann–Whitney test). (D) Number of individuals within BiKE subgroups according to stenosis degree in IgE‐sensitized and non‐sensitized groups (Fisher's exact test) and (E) IgG levels to α‐Gal (Kruskal–Wallis test with Dunn's multiple comparison test) in BiKE subgroups divided per stenosis degree. * denotes *P* < 0.05.

We further investigated whether IgE sensitization to α‐Gal was associated with specific phenotypic characteristics of carotid stenosis. The qualifying symptoms for CEA (stroke, transient ischemic attack; TIA or amaurosis fugax; AFX) were similar between the α‐Gal‐sensitized and non‐sensitized groups (Table 1). The degree of carotid stenosis did not differ between the α‐Gal sensitized and non‐sensitized groups (*p* = 0.084, Figure 1D), and the α‐Gal‐specific IgG levels were similar across different degrees of stenosis (*p* = 0.86, Figure 1E). Our findings contrast with previous studies suggesting an association between IgE and IgG to α‐Gal and increased atheroma burden. Several factors may account for these discrepant results. In the US cohort, the association between atheroma burden and α‐Gal sensitization was driven by patients younger than 65 years, whereas most BiKE patients were older than 65 (median age 72). However, we observed no age difference at CEA between α‐Gal–sensitized and non‐sensitized patients in BiKE. This suggests that sensitization is not linked to an earlier need for surgical intervention. Furthermore, disease severity might be a key determinant, and sensitization may influence disease initiation and progression: BiKE consists of patients with a severe and end‐stage form of atherosclerosis undergoing CEA, whereas the US and Australian cohorts included individuals undergoing medically warranted assessment for coronary artery disease. It may also depend on the IgE levels to α‐Gal: the median α‐Gal IgE level in the US cohort was 6.7 kUA/L [2], while in BiKE only 0.4 kUA/L. Other contributing factors may include dietary habits, genetic background, variation in co‐morbidity, and population‐specific characteristics.

**TABLE 1:** Comparison of α‐Gal sensitized and non‐sensitized groups from the cohort of carotid stenosis, BiKE.

	α‐Gal sensitized, *n* = 18	α‐Gal non‐sensitized, *n* = 82	*p*
Age, y, median (range)	72.5 (60–86)	72 (47–88)	0.277
Female, *n* (%)	2 (11.1)	35 (42.7)	0.014*
BMI (median)	24.68	25.83	0.274
Statin use, *n* (%)	12 (66.7)	59 (72.0)	0.775
Diabetes mellitus, *n* (%)	4 (22.2)	21 (25.6)	> 0.999
Smoking history, *n* (%)	11 (61.1)	64 (78.0)	0.144
Total IgE, kU/L, median (range)	52.55 (11.0–880.0)	37.58 (0–1300.0)	0.287
α‐Gal‐specific IgE, kUA/L, median (range)	0.40 (0.12–6.76)	< 0.1	—
Ratio IgE to α‐Gal/total IgE, median (range)	0.011 (0.001–0.065)	—	—
α‐Gal‐specific IgG, mg/L, median (range)	3.53 (2.1–20.1)	< 2.0 (< 2.0–11.58)	< 0.0001
Stroke, TIA, AFX, *n* (%)	9 (50), 4 (22.2), 5 (27.8)	39 (47.6), 21 (25.7), 21 (25.7)	> 0.999

*Note:* * denotes *P* < 0.05.

A limitation of the study is that the control cohort was smaller than the disease cohort (27 vs. 100 subjects), which may reduce the precision of control estimates and limit statistical power. Nevertheless, the use of nonparametric methods and the reporting of confidence intervals support the robustness of the observed group difference.

Thus, in this first European study investigating anti‐α‐Gal antibody responses in patients with end‐stage carotid stenosis, an association was noted between α‐Gal‐specific IgG levels and the presence of the disease. However, no association was observed between IgE sensitization to α‐Gal and either the presence of the disease or phenotypic characteristics of carotid stenosis. Further studies are essential to delineate the potential role of α‐Gal‐specific antibody responses in early‐stage carotid atherosclerosis by incorporating younger age groups and a broader range of disease severities.

## Author Contributions

M.P., D.A., and M.v.H. designed the study. U.H. and A.S.‐S. provided the patients' material. M.P. performed the experiments together with M.R. as well as with J.G. and M.K. M.P. analyzed and interpreted the data together with M.v.H. and U.H. M.P. and M.v.H. wrote the first draft of the manuscript. All authors critically reviewed the manuscript and approved the submitted version.

## Funding

This work was supported by The Swedish Heart‐Lung Foundation (grant numbers 2024056325, 20180036, 20200531, 2023044, 20210085 and 20220143), the Swedish Research Council (grant numbers 2017‐01070 and 2021‐01516), Region Stockholm (ALF project FoUI‐986234), The Swedish Asthma and Allergy Association's Research Foundation (grant number F2022‐0011, F2025‐0041), The Bill and Melinda Gates Foundation, The Swedish Cancer and Allergy Foundation, The Hesselman Foundation, The King Gustaf V 80th Birthday Foundation, Konsul Th C Bergh Foundation, Tore Nilsons Foundation, Magnus Bergvall Foundation, King Gustav Vth and Queen Victoria's Foundation, and MedTechLabs.

## Ethics Statement

The study was approved by the local ethics committee (Ethical permit 02‐147 and 2015/1949‐31/4) and performed in accordance with the Declaration of Helsinki.

## Conflicts of Interest

M.v.H. has received lecture fees from Thermo Fisher Scientific, Astra Zeneca, and ALK outside the submitted work. The rest of the authors report no conflicts of interest.

## Supporting information


**Table S1:** Demographic and clinical characteristics of BiKE and control cohort.

## Data Availability

The data that support the findings of this study are available from the corresponding author upon reasonable request.
