# Supplementary material for: IgE And IgG Antibody Responses to α‐Gal and Association With Carotid Stenosis in Swedish Patients
Source: Allergy. 2026 Apr 12;81(8):2966–8. doi: 10.1111/all.70329 (PMC13465965; doi:10.1111/all.70329)
Supplement: Supplementary file 1 [file ALL-81-2966-s001.docx]

IgE and IgG antibody responses to α-Gal and association with carotid stenosis in Swedish patients

Marija Perusko^1,2^, Danijela Apostolovic^1,2^, Jeanette Grundström^1,2^, Malin Kronqvist^3^, Mirjana Radomirovic^4^, Anna Smed-Sörensen^1,2^, Ulf Hedin^3^, Marianne van Hage^1,2^

^1^Division of Immunology and Respiratory Medicine, Department of Medicine Solna, Karolinska Institutet and Karolinska University Hospital, Solna Sweden

^2^ Center for Molecular Medicine, Karolinska University Hospital, Solna, Sweden

^3^ Department of Molecular Medicine and Surgery, Karolinska Institutet and Karolinska University Hospital, Sweden

^4^ University of Belgrade – Faculty of Chemistry, Belgrade, Serbia

A control cohort consisted of patients with acute respiratory viral infection without cardiovascular disease (n=27). Patients from both BiKE (n=100) and control cohort, resided within the Stockholm greater area, an area geographically overlapping with the tick *I. ricinus* infestation area. Demographic and clinical characteristics are presented in Table S1.

Table S1. Demographic and clinical characteristics of BiKE and control cohort.

|  | BiKE | Control cohort | P value |
| --- | --- | --- | --- |
| Age, y, median (range) | 70.7 (47 – 88) | 69.0 (48 – 89) | 0.15 |
| Female, n (%) | 37 (37%) | 11 (41%) | 0.824 |
| Total IgE, kU/L, median (range) | 41 (<2 – 1300) | 60 (<2 – 862) | 0.215 |
| α-Gal-specific IgE, kU_A_/L, median (range) | <0.1 (<0.1 – 6.8) | <0.1 (<0.1 – 1.4) | 0.593 |
| α-Gal-specific IgG, mg/L, median (range) | <2 (<2 – 20.1) | <2 (<2 – 9.5) | 0.024* |

**Statistical analysis**

Data were analyzed using the GraphPad Prism software, version 10.6 (La Jolla). Fisher’s exact test was used for comparisons of the categorical variables between the groups. Non-normally distributed continuous data were compared by the Mann–Whitney test to assess whether the distributions between two independent groups differ. The Kruskal-Wallis test followed with Dunn’s multiple comparison test was used for comparisons across four independent groups.
